# Association of Dietary Calcium Intake with Dental, Skeletal and Non-Skeletal Fluorosis among Women in the Ethiopian Rift Valley

**DOI:** 10.3390/ijerph19042119

**Published:** 2022-02-14

**Authors:** Demmelash Mulualem, Dejene Hailu, Masresha Tessema, Susan Joyce Whiting

**Affiliations:** 1 School of Human Nutrition and Food Science, Hawassa University, Hawassa P.O. Box 5, Ethiopia; solymico27@gmail.com; 2 School of Public Health, Hawassa University, Hawassa P.O. Box 5, Ethiopia; dejenkassa@yahoo.com; 3 Ethiopian Public Health Institute, Addis Ababa 1242, Ethiopia; masresha88@gmail.com; 4 College of Pharmacy and Nutrition, University of Saskatchewan, Saskatoon, SK S7N 5E5, Canada

**Keywords:** calcium intake, fluoride, dental fluorosis, skeletal fluorosis, non-skeletal fluorosis, Ethiopian Rift Valley

## Abstract

Fluorosis is a major public health problem in the Rift Valley of Ethiopia. Low calcium (Ca) intake may worsen fluorosis symptoms. We assessed the occurrence of fluorosis symptoms among women living in high-fluoride (F) communities in South Ethiopia and their associations with dietary Ca intake. Women (*n* = 270) from two villages provided clinical and questionnaire data. Dental fluorosis examination was done using Dean’s Index, and skeletal and non-skeletal fluorosis assessment was carried out using physical tests and clinical symptoms. Daily Ca intake was estimated by a food frequency questionnaire. Food, drinking water and beverage samples were analyzed for F level. Many subjects (56.3%) exhibited dental fluorosis. One-third of the women were unable to perform the physical exercises indicative of skeletal fluorosis; about half had ≥2 symptoms of skeletal/non-skeletal fluorosis. The average F level in drinking water sources was ~5 mg/L. The F content in staple food samples varied from 0.8–13.6 mg/kg. Average Ca intake was 406 ± 97 mg/day. Women having ≤400 mg/day Ca intake had ~3 times greater odds of developing skeletal rigidity with joint pains [AOR = 2.8, 95%CI: 1.6, 5.0] and muscular weakness [AOR = 2.9, 95%CI: 1.3, 6.3] compared to those with higher intakes. No association of calcium intake was seen with dental fluorosis. As low dietary Ca intake was associated with symptoms related to skeletal and non-skeletal fluorosis, this warrants nutritional intervention on calcium intakes in this setting.

## 1. Introduction

Drinking water sources in the Rift Valley of Ethiopia contain fluoride (F) levels exceeding the World Health Organization (WHO) limit of 1.5 mg/L [1,2]. As a consequence, many people living in the region are severely affected by fluorosis [2,3,4,5,6,7,8]. In serious cases, dental fluorosis is manifested as brown mottling of the enamel and results in overall yellowing of the teeth with erosion of the enamel [2,8]. Skeletal fluorosis occurs when there is a high degree of bone brittleness due to excess deposition of F in bone that leads to osteosclerosis [3,6,9,10,11,12]. Non-skeletal fluorosis includes muscle weakness manifestations, such as stiffness of the back and neck muscles and pain leading to inability to carry out routine domestic activities [2,9].

Fluorosis is a major public health concern particularly for developing countries including Ethiopia, as the infrastructure needed to remove the excess F ions is lacking or not widely accepted [13,14]. It can affect both children and adults [13]. Over 85% of Ethiopians living in the Rift Valley have been exposed to excess levels of F intake [7,15,16]. Although drinking water is the dominant pathway of F exposure in Ethiopian Rift Valley areas, food stuffs prepared with high-F cooking water is also an additional pathway [17]. Foods added an average of 2.3 to 4.8 mg/kg F depending on village location and type of foods consumed, both plant and animal foods are locally grown in this high-F environment [2,17].

Epidemiological studies of communities with similar F exposures have shown a relationship between calcium (Ca) intake and reduction in severity of dental fluorosis [2,17,18,19]. The Ca binds with F forming an insoluble Ca fluoride complex in the gastrointestinal tract, preventing absorption and reducing the extent of F exposure [20]. In this way the adverse effects of F are decreased. Two animal studies have shown that proof of principle [20,21]. To our knowledge, only one study has reported an inverse association between dietary Ca (milk) and severity of dental fluorosis in individuals in Ethiopia [4] and no one has reported the association between Ca intake and the severity of skeletal and non-skeletal fluorosis.

In many areas of Ethiopia, dietary Ca intake, particularly of child-bearing women, is well below the recommended dietary allowance [22,23]. F exposure may be an added concern for women’s bone and dental health where there is low Ca intake. Therefore, the purpose of this study was to assess the dental, skeletal and non-skeletal fluorosis symptoms and associations with dietary Ca intake among women of child-bearing age in the Ethiopian Rift Valley.

## 2. Materials and Methods

### 2.1. Study Design

A community-based cross-sectional study design was conducted in two purposively selected kebeles (small administrative divisions of a district, also called villages). The women included in this cross-sectional study were eligible for a planned pilot trial on eggshell supplementation [23] as a Ca source to mitigate fluorosis symptoms [20,21]. The kebeles were known for having fluorosis problems [2].

### 2.2. Setting

The two kebeles included were situated in Halaba district which is located in the main Rift Valley of Southern Ethiopia. The selected two kebeles were known for using drinking water sources with high levels of fluoride content [2]. Halaba is situated 1800 m above sea level and has low average annual rainfall (750 mm), which is the major limiting factor of agricultural production. The district is one of the districts in southern Ethiopia where drought often occurs.

### 2.3. Inclusion and Exclusion Criteria

The participant inclusion criteria were being biological mothers of young children (6–18 months) who were subjects of a parallel study, as well as being permanent residents in the selected kebeles. Mothers were excluded if they were taking drugs for any type of illness, had any apparent signs and symptoms of fever and cough at the time of enrollment and those enrolled in any health and nutritional intervention program.

### 2.4. Sample Size and Sampling

The sample size was estimated using EpiInfo Version 7.0.8.3 for descriptive studies by considering 12% prevalence of clinical symptoms of skeletal and non-skeletal fluorosis among school-age children in the same study area [2], 95% level of confidence, an absolute precision, (d = 5%) and a design effect of 1.5 (to account for cluster sampling). Finally, based on the above assumptions and considering 10% non-response rate, a minimum sample size of 270 mothers were recruited to determine the level of skeletal and non-skeletal fluorosis among women.

A list of mothers of young children aged 6–18 months old was obtained from the kebeles health post. Using this list, a house-to-house registration was conducted by giving an exclusive code number for each household, and a total of 301 eligible participants were identified in the two kebeles. Finally, a total of 270 eligible women were included in this study.

### 2.5. Variables, Data Collection and Measurements

The dependent variables for this study were dental, skeletal and non-skeletal fluorosis symptoms. The main exposure variable was dietary Ca intake. Other predictor variables included: age, educational status, occupation, income, parity, diet diversity, drinking water source, water treatment, family size and years of residency. Data on socio-demographic and economic characteristics, water supply, hygiene and environmental sanitation and obstetric history of mothers were collected using a semi-structured questionnaire that was translated to *Hallabigna*, the language of the district. The interview was conducted face to face with the selected women by trained data collectors.

### 2.6. Dietary Measures

We collected data on specific foods consumed by women in the previous 24 h using a structured questionnaire in order to determine the dietary diversity score. The recall was repeated on 10% of women to determine if these recalls were similar when conducted on different days of the week. In order to determine an individual dietary diversity score, foods were placed into each of ten food groups. Women who consumed five or more food groups in that day were considered to have fulfilled the minimum dietary diversity [24].

A structured food frequency questionnaire was used to assess the usual dietary Ca intake of mothers by asking the daily and weekly frequencies of consumption of specific Ca-rich foods common in this district (milk, yogurt, millet, fruits, kale, other vegetables and moringa). Portion size estimation was made by providing bowls and plates to each mother to help them visualize and serve the amount of each food consumed. These frequencies were converted to daily dietary Ca intakes, as described by Gozdzik et al. [25] using published Ethiopian values for Ca content [26], and recipes were used to identify the amount of Ca in high-Ca foods, such as millet injera [27]. The assumptions used to convert frequencies of consumption to daily dietary Ca intake included: if the frequency of consumption of a particular food more than once a day assumed to be twice a day; if once per day, assumed to be once a day; if more than once per week (i.e., 3.5 days on average, thus, 3.5/7 = 0.5), so assumed as to be 0.5 times per day; if once per week (1/7 = 0.15), so assumed as to be 0.15 times per day; if once per month (1/30 = 0.033), so assumed as to be 0.033 times per day; and if not consumed over that month, assumed as not consumed daily during that monthly period [25].

### 2.7. Determination of Dental, Skeletal and Non-Skeletal Fluorosis

The women were assessed for dental fluorosis using Dean’s index [28] which classifies fluorosis on a scale of 0 to 4 as: Class 0, No Fluorosis; Class 1, Very Mild Fluorosis (opaque white areas irregularly covering 25% of the tooth surface); Class 2, Mild Fluorosis (white areas covering 25–50% of the tooth surface); Class 3, Moderate Fluorosis (all surfaces affected, with some brown spots and marked wear on surfaces subject to attrition); and Class 4: Severe Fluorosis (widespread brown stains and pitting). The average score of the two most severely affected teeth was used to derive this classification. The women were instructed to thoroughly clean their teeth prior to the dental fluorosis examination. The dental assessment was conducted by an experienced dentist (MD and DDS degrees) who was hired for this purpose.

Skeletal and non-skeletal fluorosis were determined using clinical symptoms and physical exercises as developed by Susheela [29] and Shashi et al. [12] and as described by Kebede et al. [2]. Individuals who could not perform the physical exercises were categorized as having skeletal fluorosis [12]. The assessment was carried out by an experienced physiotherapist (MSc degree) who was hired to perform the physical examination. All study women were asked to perform three physical exercises which indicate skeletal fluorosis [2]: (i) bending the body and touching floor or toe; (ii) touching chest with chin; and (iii) folding arms to touch back of head. The physiotherapist showed each mother how to perform the exercise. The same physiotherapist assessed the women for the presence or absence of thirteen symptoms of skeletal and non-skeletal fluorosis which are itemized below in the Results section (Table 5).

### 2.8. Food and Water Sample Collection and Analysis

The food samples were dried, grounded and homogenized using drying oven, miller and homogenizer respectively for F analysis. Aliquots of 5 mL were taken out for analysis after the ash had settled and the solutions were clear. Care was taken to avoid the settled ash. Before analyzing, 0.5 mL TISABIII was added to obtain a pH of 5.2–5.4, which is the optimal pH-range for F determination. Reagent blanks were always prepared together with the samples and were brought through the whole procedure. The F level of standard solutions and sample solutions were measured in ppm using an F ion-selective electrode. For all food samples, 1 mL of 100 ppm F standard was spiked to determine percent recovery. Water F was determined by using ES ISO 10359–1: 2001 method, whereas food F level was measured by food F test method [17]. The electrode was calibrated by working standards starting from most diluted to concentrated standard. The concentrations of the solutions were directly measured in ppm by using an F ion-selective electrode.

### 2.9. Bias, and Data Management

Effort was made to address possible potential sources of information bias. After setting exclusion criteria, we included all eligible women. In order to minimize information bias, the dental, skeletal and non-skeletal fluorosis assessment was conducted by a qualified dentist and physiotherapist who were hired for this purpose. These specialists used pre-defined and validated assessment tools to arrive at a presumptive diagnosis of the condition.

All quantitative data were double-entered in to EpiData version 3.1, and then exported into SPSS version 25 for analyses. All continuous data were checked for normal distribution using the Kolmogorov–Smirnov Test of Normality. Collinearity was checked using variance inflation factor (<10) and tolerance (>0.1), and there was no multicollinearity between the independent variables [30].

### 2.10. Fluorosis Symptom Indices

Data on skeletal and non-skeletal fluorosis symptoms were categorized into three symptom indices according to Tandon et al. [9]. Index-1 is named skeletal rigidity and joint pain, Index-2 is named muscular weakness manifestations and Index-3 is named gastro-intestinal complaints. Index-1 included: skeletal rigidities and pain of the neck region, back, knee and shoulder joints. Index-2 included: stiffness of the back and neck muscles, unable to bend forward and to stand straight and inability to carry out routine activities. Index-3 included: early signs of F toxicity, such as loss of appetite, nausea, pain in the stomach, constipation and gas formation in the stomach with a bloated feeling. When a woman complained of ≥2 symptoms of these types of skeletal and non-skeletal fluorosis symptoms, she was assigned a score of 1 and if not, scored 0. This was carried out for each fluorosis index. Data on dental fluorosis were analyzed as 1 = having any level of dental fluorosis severity (very mild through severe) and 0 = if questionable or normal. The dichotomized value for each index as well as for dental fluorosis was used in the regression analysis.

### 2.11. Statistical Methods

Descriptive statistics, such as mean and frequency, were used to display study results. Logistic regression analysis was conducted to identify factors that could be associated with fluorosis symptoms. The factors were first included in bivariable logistic regression models to check their association with fluorosis symptoms indices. The variables were included in multivariable logistic regression analysis if *p* < 0.2. The variables included in the multivariable logistic regression model were age of women, educational status, occupation, parity, DDS, dietary Ca intake, family size and years of residency.

### 2.12. Ethics and Informed Consent

This study was conducted according to the guidelines laid down in the Declaration of Helsinki and all procedures involving research study participants were approved by Hawassa University College of Medicine and Health Science (Ref. No: IRB/019/10) and University of Saskatchewan Biomedical Research Ethics, Saskatoon, Canada (Ref. No: Bio# 17-150). After the purpose and methods of the study were fully explained, and their right to refuse was explained, informed verbal and written consents were obtained from all study participants prior to their participation in the study.

## 3. Results

### 3.1. Socio-Demographic and Economic Characteristics

The mean (SD) age of the women in completed years was 29.5 ± 4.2 years. Most of the women (96.5%) were married, the majority (67.4%) had not attended school (were illiterate) and just more than half (51.9%) of them had no paid job outside their home. The main source of income for most (87.8%) of the households was agriculture and the majority (82.2%) of them earned an average of less than 1000 ETB birr per month (~$20 USD). The average family size (parents and children) was approximately 7 ± 2 (Table 1).

### 3.2. Water Supply, Hygiene and Environmental Sanitation

The main source of drinking water for all households was a tap at a common public pipe, and the majority (63.7%) of the study participants walked about an hour to get the drinking water from the public pipe. Most of the study participants (82.2%) were not filtrating or treating the water before drinking it. Pit latrine was available in most (88.5%) households among the study women and most (89.3%) of them used a bar of soap while washing their hands (Table 2).

There was no attempt by study participants or health workers in these kebeles to take any measure (including dietary) to lessen the toxic effects of excess F intake. Study participants also did not receive health education about excess intake of F in foods or drinks, its consequences on health (fluorosis) and measures needed to be taken to reduce F exposure, prior to this study. None of the households used rainwater for drinking as it was scarce and due to fearing of dusts contamination from the roof. Bed nets were available in less than half (44.4%) of the study participants’ households.

### 3.3. Obstetric History of Women

Regarding the history of childbirth of the women, 23.3% of mothers had a history of spontaneous abortion (i.e., miscarriage) and 15.2% had a history of stillbirth. The average of live-born children by the study mothers was approximately four (data not shown).

### 3.4. Fluoride Level in Food, Beverage and Water Samples

The F content in staple food samples varied between 0.8–13.6 mg/kg. The foods which were prepared from maize and millet had higher F levels, whereas vegetable food sources, such as cabbage and potato had low concentration of F. For all food items, percent recovery varied between 90.4–107.9%, which falls on the acceptable range of percent recovery. All common drinking water sources in the study area had an F level exceeding the WHO guidelines for drinking water (1.5 mg/L). The average F level in drinking water sources was approximately 5 mg/L (Table 3).

### 3.5. Dietary Calcium Intake of Women

The average daily dietary Ca intake of women was 406 ± 97 mg (Table 4). Only 37.4% of women consumed one or more locally available Ca-rich foods daily. More than half (55.2%) of the women also did not consume yogurt and approximately one-quarter (23.3%) did not drink milk during the last month prior to this survey. No participant reported consuming moringa. The mean (SD) dietary diversity score of women based on 10 food groups was (mean (SD)) 4.92 (1.91).

### 3.6. Fluorosis Assessment of Women

Overall, more than half (56.3%) of the study women had very mild to severe dental fluorosis (Table 5). The percentage of severe dental fluorosis was 9.3%; and the percentage of women with moderate dental fluorosis was 25.2%.

The overall percentage of women who were unable to perform the physical exercises which indicate skeletal fluorosis was 34.4%. During the skeletal fluorosis examination, 40.4% of the women were unable to touch chest with chin and 34.8% of the women were unable to touch back of head by stretching and folding their hands. Overall, 124 (45.9%) of the women complained of more than one symptom listed in Table 5. The majority of the women felt lower back pain (70.7%) and had constipation (79.3%). More than half (54.8%) of them also had muscle weakness. In addition, around half of the women had neck pain (46.3%) or had been complaining of abdominal pain (48.5%).

### 3.7. Association of Fluorosis Indices with Dietary Ca Intake

As shown in Table 6, we tested the association of skeletal fluorosis with subject characteristics and environmental factors. For skeletal rigidity and joint pains, Index-1, age, diet diversity and dietary Ca intake were significantly associated with having two or more symptoms. For having muscular weakness, Index-2, age, parity and dietary Ca intake were significantly associated, in the adjusted model. For both Index-1 and Index-2, only age and dietary calcium contributed to worsening of this condition. This study revealed that women whose dietary Ca intake was less than 400 mg/day, which was the average intake (Table 4), had about three times greater odds of developing Index-1 skeletal rigidity with joint pains (AOR = 2.8, 95%CI: 1.6, 5.0) and muscular weakness, Index-3 (AOR = 2.9, 95%CI: 1.3, 6.3), than those who had higher intake.

The odds of developing both Index-1 and Index-2 fluorosis symptoms were 1.2 (95%CI: 1.0, 1.2) and 1.7 (95%CI: 1.5, 2.0), respectively, with each passing year of age (Table 6). No variables were significantly associated (*p* > 0.05) with either Index-3 (Gastro-intestinal complaints) (Appendix A Table A1) or dental fluorosis (Appendix A Table A2).

## 4. Discussion

More than half of the women in this study had dental fluorosis as diagnosed using Dean’s index criteria [28]. This is in line with the findings of a recent systematic review which reported a high prevalence of dental fluorosis in the Ethiopian Rift Valley [7]. In addition, a high prevalence of dental fluorosis was reported among children living in moderate F (24.1%) and high-fluoride (75.9%) areas of the Ethiopian Rift Valley [13]. Another study also reported a higher prevalence of dental fluorosis (62%) among adult inhabitants in the main Rift Valley of Ethiopia [4]. Our data show that women of child-bearing age can be affected by high F, but dental fluorosis develops mainly during early childhood [31]. In contrast with Kravchenko et al. [4], who reported an inverse association with milk intake in young adults living in the Rift Valley, we found no predictors of dental fluorosis. This could be due to the fact that dental fluorosis is an irreversible process that occurred as a result of long-term exposure to excess fluoride during childhood period, and thus was no longer related to current calcium consumption.

Few studies have reported on non-dental fluorosis symptoms in women. We classified symptoms according to skeletal rigidity and pain (skeletal fluorosis), muscular weakness (a major outcome of non-skeletal fluorosis) and gastro-intestinal complaints (other symptoms of non-skeletal fluorosis [9]. Skeletal rigidity accompanied by joint pains was common among women. Muscular weakness manifestations were also observed in many of the women. In addition, early signs of F toxicity, such as gastro-intestinal complaints manifested as loss of appetite, nausea and constipation, were observed in some of the women.

The women in the present study had a low intake of dietary Ca, averaging close to 400 mg per day. This is in agreement with the findings of Tesfaye et al. [22] who assessed national Ca intakes of Ethiopian women in the reproductive age using a single 24 h dietary recall and found the national average to be 478 mg per day. For women in the same state as our study, the Southern Nations, Nationalities and People Regional state (SNNPR), these authors found average Ca intake of 622 mg per day. In contrast, the Ca intakes of women in this study were estimated from daily and weekly consumption frequencies of Ca-rich foods during a one-month period. Differences may be due to different methodologies or actual differences in our kebeles; however, both sets of results show a lack of adequate dietary Ca in most women.

In our regression analysis, study mothers who had inadequate Ca intake (≤400 mg/d) had three times increased odds of developing skeletal and non-skeletal fluorosis symptoms, namely skeletal rigidity with joint pains and muscular weakness manifestations, compared with those with higher Ca intake. To our knowledge, no study has been conducted to assess Ca intakes as predictors of skeletal or non-skeletal fluorosis symptoms. Kravchenko and colleagues [4] found a significant correlation between milk intake and dental fluorosis severity among men and women in the Ethiopian Rift Valley. Therefore, milk was the main source of Ca and presumably measured intake represented usual practice. Not many women in our study ingested dairy products as a source of Ca.

A decrease in F absorption by dietary Ca has only been demonstrated in a small number of animal studies [20,21]. In these experiments, Ca added to the diet reduced urinary F excretion, indicating the gastrointestinal binding of F ions by Ca. Kebede et al. [20] also verified this finding by showing a decrease in fecal F levels. However, most support for the Ca effect on F has been shown in observational studies. An earlier epidemiological study of children in India reported greater prevalence of fluorosis symptoms among children with inadequate dietary Ca intake [18]. In that study, children with adequate dietary Ca (>800 mg/d) and deficient dietary Ca (<300 mg/d) having comparable intakes of F (mean 9.5 ± 1.9 mg/d) were compared; severe toxic effects of F were observed in children with Ca deficiency [18].

Similarly, our recent efficacy trial provided a proof of concept on calcium intake and fluoride. In this trial, the study subjects were supplemented with an approximate of 1000 mg of calcium (using calcium-containing eggshell powder) on a daily base for six consecutive months. The urinary F excretion in the supplemented group was six-fold lower (β = −6.1 (95% CI: −7.1, −5.1)) compared to the control group. The risk of developing skeletal and non-skeletal fluorosis was also significantly (*p* < 0.001) reduced in the treatment group [23].

Gastrointestinal complaints (Index-3) were not associated with Ca intake, or any other factors related to development of fluorosis, in contrast to skeletal rigidity and pains (Index-1) and muscular weakness (Index-2). Assefa et al. [3] used four clinical signs of non-dental fluorosis and compared these to radiological confirmation of skeletal fluorosis in 180 men and 5 women in Ethiopia from a high F area, mean age 55 years. Skeletal fluorosis was present in 70% of subjects, while clinical prevalence using kyphosis, impaired squatting, impaired neck mobility and impaired lumbar mobility, averaged 20%. This indicates clinical signs have low sensitivity; however, all signs except kyphosis were in high agreement (*p* < 0.001) with radiological skeletal fluorosis, indicating good specificity.

In the current study, older age was significantly associated with skeletal and non-skeletal fluorosis symptoms. This is in line with the findings of a community-based survey on epidemiology of skeletal fluorosis by Melaku and his colleagues [5]. These authors reported that older people of age 55 years and above had 20 times higher risk of developing skeletal fluorosis than adolescents and young adults of 15 to 24 years of age [5]. This could be due to the fact that in an area where F intake is higher, more F would be accumulated in bones and soft tissues of individuals who consumed it throughout their longer lifetime.

Our findings showed that women that have a high parity had greater odds of developing skeletal and non-skeletal fluorosis symptoms compared with those with lower parity. Older women were found to have a greater number of children, and hence parity may reflect the older age of women with more children and additionally, the stress of repeated pregnancy and lactation that may have depleted the Ca stores of these women [32,33]. Consequently, fluorosis symptoms would be manifested more among the women with high parity level. Therefore, this finding may have implications for recommendations of Ca during pregnancy and lactation in high-F areas.

The F level in drinking water sources in this area averaged ~5 mg/L and in staple foods ranged from 0.8–13.6 mg/kg. Similarly, the F level of the drinking water in this area was previously reported as 4.6 ± 1.7 mg/L [2]. The primary preferred option may be to find a supply of safe drinking water with safe F levels. In such instances, de-fluoridation may be sought as a solution [34,35]. However, there was no defluoridation of high-F containing water as well as no fluorosis prevention strategies attempted in the current study area. In this area, rainwater, a source of low-F drinking and cooking water, was not used for drinking as it was scarce and due to fearing of dust contamination from the roof of the house when available. Rainwater is not viewed as clean in many parts of the world [36]. In terms of addressing fluorosis symptoms, new technologies may assist, for example, in remineralization of teeth [37].

The limitations of this study include using some non-specific clinical symptoms and physical exercise tests to assess skeletal and non-skeletal fluorosis may be inaccurate and may introduce mis-classification bias. However, we employed the same dentist and physiotherapist to do all measurements, which reduces measurement bias. We also did not include women with only one symptom as being fluorotic to minimize the false positives arising from counting one nonspecific symptom. For skeletal fluorosis, we did not have access to x-ray measurements. We combined symptoms to create unique indices in order to analyze the presence of multiple symptoms. Daily dietary calcium intake of the women was estimated from frequencies of calcium-rich foods consumption which might not have estimated the usual calcium intake of women beyond that month.

## 5. Conclusions

Signs and symptoms of dental, skeletal and non-skeletal fluorosis were prevalent in women of child-bearing age in this area of the Rift Valley of Ethiopia, where water and food sources of fluoride were high. Dietary calcium intake was low relative to requirements, averaging only 400 mg per day. The low intake of dietary calcium, less than 400 mg per day, was significantly associated with musculo-skeletal fluorosis symptoms but not with non-skeletal fluorosis or dental fluorosis. The presence of musculo-skeletal fluorosis impairs everyday life for the women, who have a very labor-intensive life of raising children and having farm and home responsibilities. This suggests the need for further investigation on improving calcium intakes to mitigate the toxic effects of high fluoride intake in the Ethiopian Rift Valley.

## Figures and Tables

**Table 1:** Socio-demographic and economic characteristics of study participants in Halaba district, southern Ethiopian Rift Valley, 2018.

Characteristics (*n* = 270)		*N*	(%)
Mother’s educational status	Illiterate (hadn’t attended school)	182	67.4
	Primary education (1st to 8th grade)	74	27.4
	Secondary education (9th to 12th grade)	11	4.1
	College (above 12th grade)	3	1.1
Mother’s main occupation	No outside job/housewife/	140	51.9
	Agriculture	96	35.6
	Petty trading	29	10.7
	Employed	5	1.9
Main source of household income	Agriculture	237	87.8
	Petty trading	24	8.9
	Employed	9	3.3
Estimated average monthly income of the household (ETB) ^1^	<500	115	42.6
	501–1000	107	39.6
	1001–1500	26	9.6
	1501–2000	9	3.3
	>2000	13	4.8
Residency	>15 years	138	51.1
	11–15 years	46	17.0
	5–10 years	62	23.0
	<5 years	24	8.9

^1^ ETB = Ethiopian birr. 1 ETB = $0.02USD in 2018.

**Table 2:** Water, hygiene and environmental sanitation of study participants in Halaba district, southern Ethiopian Rift Valley, 2018.

Characteristics (*n* = 270)		*N*	%
Time to bring water from public pipe	1 h or less	172	63.7
	>1 h	98	36.3
Equipment used to store drinking water	Jerrican	215	79.6
	Bucket	55	20.4
Filtrating/treating the water before drinking	No	222	82.2
	Yes	48	17.8
If yes, method of filtration (*n* = 48)	Water treatment pills	29	60.4
	Filtering cloths	17	35.4
	Boiling and removing sediment	2	4.2
Use same water source for drinking and cooking	No	230	85.2
	Yes	40	14.8
Use same water source for drinking and bathing	No	220	81.5
	Yes	50	18.5
Knowledge on hand washing (affirmative)	Before preparing food	42	15.6
	Before feeding the child	226	83.7
	After using toilet	215	79.6
	After cleaning child (defecated)	201	74.4
Substance used for hand washing	Soap bar	241	89.3
	Just only water	20	7.4
	Liquid soap	9	3.3
Household latrine type	Outside home latrine	239	88.5
	Protected open field	20	7.4
	No latrine (open field)	11	4.1

**Table 3:** Fluoride level in staple foods, beverage and drinking water consumed by the study subjects in Halaba district, southern Ethiopian Rift Valley, 2018.

Sample Type	Fluoride	Recovery (%)
Food	mg/kg	
Injera (unknown ingredients)	10.6	100.6
Unleavened bread (unknown ingredients)	13.6	97.2
Unleavened bread, maize	12.5	102.5
Taro, Colacasia antiquorum:, boiled	4.6	96.2
Injera (Maize, Millet (1:1))	8.3	102.5
Unleavened bread, millet	4.9	93.7
Boiled cabbage	0.8	103.2
Boiled potato and carrot	1.6	92.2
Stew without tomato	3.9	103.6
Banana	5.3	107.9
Boiled beetroot	3.2	102.7
Cabbage and potato stew	2.4	95.9
Rice with carrot	3.2	103.6
Injera (sorghum, maize, millet (1:1:1))	4.9	95.4
Injera (millet)	7.8	105.7
Bread (maize)	10.5	99.0
Beverage	mg/kg	
Coffee (prepared from coffee leaf)	3.5	104.5
Drinking water	mg/L	
Drilled tap water	4.7	
Hand pump water	6.2	
Well water	3.3	

**Table 4:** Frequency of calcium-rich food consumption by women in Halaba district, southern Ethiopian Rift Valley, 2018.

Frequency of Consumption (*n* = 270)	Milk *N* (%)	Yogurt *N* (%)	Millet *N* (%)	Kale *N* (%)	Fruits * N (%)	Other Vegetables ^#^ *N* (%)	Moringa *N* (%)
>Once per day	29 (10.7)	21 (7.8)	114 (42.2)	125 (46.3)	22 (8.1)	25 (9.3)	0
Once a day	27 (10.0)	19 (7.0)	66 (24.4)	61 (22.6)	9 (3.3)	12 (4.4)	0
>Once per week	32 (11.9)	15 (5.6)	55 (20.4)	49 (18.1)	39 (14.4)	38 (14.1)	0
Once a week	67 (24.8)	30 (11.1)	26 (9.6)	35 (13.0)	147 (54.4)	122(45.2)	0
Once a month	52 (19.3)	36 (13.3)	9 (3.3)	0	16 (5.9)	25 (9.3)	0
Not consumed	63 (23.3)	149 (55.2)	0	0	37 (13.3)	48 (17.8)	270 (100)
Daily dietary calcium (mg): 406.4 ± 96.8							

**Table 5:** Prevalence (%) of fluorosis among women (n = 270) in Halaba district, southern Ethiopian Rift Valley, 2018.

Measurement	N	%
Dental fluorosis category		
Normal	96	35.6
Questionable	22	8.1
Very mild: opaque white areas covering 25% of the tooth surface	38	14.1
Mild: white areas covering 25%–50% of the tooth surface	21	7.8
Moderate: all surfaces affected, with some brown spots and marked wear on surfaces subject to attrition	68	25.2
Severe: widespread brown stains and pitting	25	9.3
Skeletal Fluorosis Exercise Testing		
Unable to bend body and touch floor or toe	76	28.1
Unable to touch chest with chin	109	40.4
Unable to stretch and fold arms to touch back of head	94	34.8
Symptoms of Non-skeletal Fluorosis		
Feel lower back pain	191	70.7
Feel leg pain, joints	182	67.4
Feel arm pain, joints	143	53.0
Feel tingling in the hands and feet	172	63.7
Feel neck pain with movement	125	46.3
Feel muscle weakness	148	54.8
Feel loss of appetite	100	37.0
Have nausea	101	37.4
Have abdominal pain	131	48.5
Have bloating in stomach	60	22.2
Experience polydipsia (excessive thirst)	22	8.1
Experience polyuria (excess urine volume)	16	5.9
Experience constipation	214	79.3

**Table 6:** Factors associated with muscular-skeletal symptoms of fluorosis Index-1 and Index-2 among women: logistic regression model.

Variable (*n* = 270)	Category	Skeletal Rigidity and Joint Pains Index-1		COR (95%CI) ^#^	AOR (95%CI)	Muscular Weakness Index-2		COR (95%CI) ^#^	AOR (95%CI)
		No *N* (%)	Yes *N* (%)			No *N* (%)	Yes *N* (%)		
Age (y)	Mean (SD)	29.5 (4.2)		1.2 (1.1, 1.2)	1.2 (1.0, 1.2) *	29.5 (4.2)		1.8 (1.6, 2.0)	1.7 (1.5, 2.0) *
Education	Unable to read and write	47 (33.8)	92 (66.2)	1.6 (0.9, 2.5)	1.6 (0.9, 2.7)	65 (46.8)	74 (53.2)	1.7 (1.0, 2.7)	1.2 (0.6, 2.5)
	Able to read and write	58 (44.3)	73 (55.7)	1		78 (59.5)	53 (40.5)	1	
Occupation	No job	40 (39.6)	61 (60.4)	1		47 (46.5)	54 (53.5)	1	
	Have job	65 (38.5)	104 (61.5)	1.1 (0.6, 1.7)	1.3 (0.8, 2.4)	96 (56.8)	66 (42.3)	1.4 (0.9, 2.3)	1.5 (0.7, 3.0)
Parity	≤4 live births	32 (37.2)	54 (62.8)	1		90 (57.7)	66 (42.3)	1	
	>4 live births	73 (39.7)	111(60.3)	1.1 (0.7, 1.9)	1.2 (0.6, 2.2)	53 (46.5)	61(53.5)	1.6 (1.0, 2.6)	2.5 (1.1, 5.6) *
Family size ^$^	≤5 members	32 (45.1)	39 (54.9)	1		46 (64.8)	25 (35.2)	1	
	>5 members	73 (36.7)	126 (63.3)	1.4 (0.8, 2.4)	1.3 (0.7, 2.4)	97(48.7)	102 (51.3)	1.9 (1.1, 3.4)	1.1 (0.4, 2.3)
Diet Diversity	≤5 food groups	53 (32.5)	110 (67.5)	1.9 (1.19, 3.2)	2. 0 (1.1, 3.4) *	81 (49.7)	82 (50.3)	1.4 (0.9, 2.3)	1. 5 (0.7, 3.0)
	>5 food groups	52 (48.6)	55 (51.4)	1		62 (57.9)	45 (42.1)	1	
Dietary Ca	≤400 mg/day	35 (25.4)	103 (74.6)	3.3 (2.0, 5.6)	2.8 (1.6, 5.0) *	54 (39.1)	84 (60.9)	3.2 (1.9, 5.3)	2.9 (1.3, 6.3) *
	>400 mg/day	70 (53.0)	62 (47.0)	1		89 (67.4)	43 (32.6)	1	
Residency	≤10 years	40 (46.5)	46 (53.5)	1		57 (66.3)	29 (33.7)	1	
	>10 years	65(35.3)	119 (64.7)	1.6 (0.9, 2.7)	1.1 (0.6, 2.0)	86 (46.7)	98 (53.3)	2.2 (1.3, 3.8)	1.8 (0.8, 4.0)

^$^ Number of children and parents living together; **^#^** in the single logistic regression, all these variables were significant predictors at *p* < 0.2; * statistically significant association observed at *p* < 0.05. COR = Crude Odds Ratio AOR = Adjusted Odds Ratio.

## Data Availability

Data will be made available upon request.

## Appendix A

**Table A1:** Factors associated with non-skeletal symptoms of fluorosis Index-3 among women: a single and multivariable logistic regression model.

Variable (n = 270)	Category	Gastro-Intestinal Complaints Index-3		COR (95%CI) ^#^	AOR (95%CI) *
		No Number (%)	Yes Number (%)		
Age (year)	Mean (SD)	29.5 (4.2)		0.95 (0.89, 1.01)	0.95 (0.88, 1.01)
Education	Unable to read and write	82 (59.0)	57 (41.0)	1.1 (0.69, 1.84)	1.2 (0.72, 2.01)
	Able to read and write	81 (61.8)	50 (38.2)	1	
Occupation	No job	64 (63.4)	37 (36.6)	1	
	Have job	99 (58.6)	70 (41.4)	1.2 (0.74, 2.03)	1.2 (0.72, 2.04)
Parity	≤4 live births	92 (59.0)	64 (41.0)	1	
	>4 live births	71 (62.3)	43(37.7)	1.2 (0.70, 1.89)	1.1 (0.65, 1.91)
Family size	≤5	40 (56.3)	31 (43.7)	1	
	>5	123(61.8)	76 (38.2)	1.3 (0.72, 2.17)	1.3 (0.73, 2.33)
Diet Diversity	≤5 food groups	99 (60.7)	64 (39.3)	0.96 (0.59, 1.58)	1. 0 (0.61, 1.70)
	>5 food groups	64 (59.8)	43 (40.2)	1	
Dietary Ca	≤400 mg/day	84 (60.9)	54 (39.1)	1.1 (0.64, 1.70)	1.0 (0.58, 1.74)
	>400 mg/day	79 (59.8)	53 (40.2)	1	
Residency	≤10 years	56 (65.1)	30 (34.9)	1	
	>10 years	107(58.2)	77 (41.8)	1.3 (0.79, 2.29)	1.5 (0.84, 2.58)

**^#^** In the single logistic regression, some of variables were significant predictors at *p <* 0.2. * There was no statistical significant association observed for any of the variables at *p* > 0.05.

**Table A2:** Factors associated with dental fluorosis among women: a single and multivariable logistic regression model.

Variable (n = 270)	Category	Dental Fluorosis ^1^		COR (95%CI) ^2^	AOR (95%CI) ^3^
		Absent Number (%)	Present Number (%)		
Age (year)	Mean (SD)	29.5 (4.2)		1.0 (0.96, 1.07)	1.0 (0.96, 1.08)
Education	Unable to read and write	57 (41.0)	82 (59.0)	1.3 (0.77, 2.03)	1.2 (0.73, 1.99)
	Able to read and write	61 (46.6)	70 (53.4)	1	
Occupation	No job	37 (36.6)	64 (63.4)	1	
	Have job	81 (47.9)	88 (52.1)	1.6 (0.96, 2.64)	1.6 (0.95, 2.68)
Parity	≤4 live births	65 (41.7)	91 (58.3)	1	
	>4 live births	53 (46.5)	61 (53.5)	1.2 (0.75, 1. 98)	1.3 (0.78, 2.02)
Family	≤5	32 (45.1)	39 (54.9)	1	
	>5	86 (43.2)	113 (56.8)	1.1 (0.63, 1.86)	1.0 (0.78, 2.27)
Diet Diversity	≤5 food groups	72 (44.2)	91 (55.8)	1.1 (0.64, 1.72)	1. 0 (0.60, 1.66)
	>5 food groups	46 (43.0)	61 (57.0)	1	
Dietary Ca	≤400 mg/day	61 (44.2)	77 (55.8)	1.0 (0.64, 1.69)	1.3 (0.76, 2.24)
	>400 mg/day	57 (43.2)	75 (56.8)	1	
Residency	≤10 years	40 (46.5)	46 (53.5)	1	
	>10 years	78 (42.4)	106 (57.6)	1.2 (0.71, 1.98)	1.2 (0.68, 2.26)

**^1^** Dental fluorosis was ‘absent’ if the teeth were examined as normal or questionable; ‘present’ if the teeth were examined as very mild, mild, moderate or severe dental fluorosis. ^2^ In the single logistic regression, some of variables were significant predictors at *p* < 0.2. ^3^ There was no statistically significant association observed for any of the variables at *p* > 0.05.
